# Provider documentation of electronic nicotine delivery systems use among patients prescribed contraception at an academic health center in the Southeastern United States

**DOI:** 10.1016/j.pmedr.2021.101632

**Published:** 2021-11-11

**Authors:** Joanna M. Theophilopoulos, Jennifer H. LeLaurin, Maribeth Williams, Melissa Bright, Lindsay A. Thompson, Ramzi G. Salloum

**Affiliations:** a College of Medicine, University of Florida, Gainesville, FL, USA; b Department of Health Outcomes and Biomedical Informatics, College of Medicine, University of Florida, USA; c Department of Community Health and Family Medicine, College of Medicine, University of Florida, USA; d Department of Obstetrics and Gynecology, College of Medicine, University of Florida, USA; e Department of Pediatrics, College of Medicine, University of Florida, USA

**Keywords:** Electronic cigarettes, Vaping, Hormonal contraception, Electronic health record

## Abstract

**Purpose:**

Women on combined hormonal contraception (CHC) who use electronic nicotine delivery systems (ENDS) may be vulnerable to adverse cardiovascular events. To date, no study has examined whether clinicians screen for ENDS use when prescribing CHC. Therefore, we investigated documentation of ENDS screening and counseling in the electronic health record (EHR) of women prescribed CHC.

**Methods:**

We conducted a retrospective EHR review and content analysis at an academic health center in the Southeastern United States. We randomly selected 500 records of female patients 12 years and older who had been prescribed contraception and had ENDS documented in their records identified via keyword match. Records prior to July 2020 were reviewed between June-September 2020.

**Results:**

Of the 500 patients, 245 (49%) were ENDS users and 227 (45.4%) were non-ENDS users. Among ENDS users, there were 82 contraception-related encounters with ENDS documentation. In 55 (67.1%) of these encounters, only ENDS use status was documented. The provider counseled against ENDS use in 17 (20.7%) records. Six (7.3%) notes documented provision of patient education materials instructing patients on contraception to refrain from using ENDS. Among non-ENDS users, there were 43 contraception-related encounters with ENDS documentation; 35 (81.4%) documented the patient did not use ENDS and 3 (7%) documented provision of patient education materials.

**Conclusion:**

ENDS use is under-documented in contraception-related encounters. Improvements in documentation may help assess long-term effects of concurrent ENDS and CHC use. These results illustrate the need to clarify EHR prompts and increase provider awareness to improve ENDS documentation.

## 1 Introduction

Electronic nicotine delivery systems (ENDS) are devices that heat nicotine-containing liquid and produce an aerosol that users inhale. ENDS have been marketed as a tool to quit smoking and a safer alternative to cigarettes. ENDS use has increased exponentially over the past decade, especially among adolescents. From 2017 to 2019, there was a 135% increase in reported ENDS use among high schoolers (Cullen et al., 2019). The National Health Interview Survey found a 46.2% increase from 2014 to 2018 in ENDS use among adults aged 18–24 (Dai and Leventhal, 2019). While ENDS are purported to be safer alternatives to cigarettes, the long-term consequences are not well understood.

Given the novelty of ENDS, there is insufficient data to assess their long-term cardiovascular effects. However, several studies have observed elevations in biomarkers predictive of cardiovascular disease after ENDS exposure, including increased sympathetic response, oxidative stress, endothelial dysfunction, and platelet activation (Middlekauff, 2020). Nicotine activates the sympathetic nervous system, which increases heart rate, blood pressure, and coronary vasoconstriction (Benowitz and Burbank, 2016). Glycerol, propylene glycol, particulate matter, flavorings, and heavy metals in ENDS form reactive oxygen species that cause endothelial dysfunction and inflammation, platelet activation, and direct cardiotoxic effects (Kennedy et al., 2019).

One population that could be vulnerable to potential cardiovascular effects of ENDS is women who use combined hormonal contraceptives (CHC). Women on CHC exposed to ENDS had significantly adverse changes in vitamin E (an antioxidant), 8-*iso*-prostaglandin F2a-III (a marker of oxidative stress), and flow-mediated dilation (a marker of endothelial dysfunction) (Mastrangeli et al., 2018). These findings suggest ENDS exposure among CHC-prescribed women could lead to inflammation and endothelial dysfunction that increase the risk of cardiovascular disease. This is notable because CHC alone increases the risk of adverse cardiovascular events, particularly venous thromboembolism (Tchaikovski and Rosing, 2010, Trenor et al., 2011). Estrogen in CHC alters several proteins involved in coagulation, which can promote a hypercoagulable state (Trenor et al., 2011). The relative risk of thrombosis for women on CHC is 3–5-fold higher than women not prescribed CHC (Trenor et al., 2011).

Proper counseling can help decrease the incidence of adverse cardiovascular events among CHC-prescribed women. Because smoking is a well-established risk factor for cardiovascular disease, the use of CHC can be contraindicated in certain women who smoke (Curtis et al., 2016). A smoker younger than 35 can generally use CHC. For patients over 35 who smoke < 15 cigarettes per day, the use of CHC is usually not recommended unless there are no other contraception options for the patient. The use of CHC in patients over 35 who smoke > 15 cigarettes per day is typically contraindicated. If a patient is younger than 35 and has multiple risk factors for cardiovascular disease, CHC use may be contraindicated. Notably, these guidelines do not address ENDS use.

To date, no studies have examined whether women who use CHC and ENDS have an increased risk of cardiovascular disease (Riley et al., 2016). A fundamental problem may lie in the fact that provider awareness and documentation practices have lagged behind the rise of ENDS use. The objective of this study was to examine ENDS documentation practices in the electronic health records (EHRs) of women prescribed contraception. We sought to investigate whether healthcare providers screen for and counsel patients on ENDS use when they prescribe contraception.

## 2 Materials & Methods

This study was an exploratory retrospective review of EHRs from an academic health center in the Southeastern U.S. that uses the Epic EHR system. Records from multiple specialties and clinical settings were reviewed, including inpatient and outpatient OB-GYN, pediatrics, family medicine, internal medicine, and surgical subspecialties. Records prior to July 2020 were eligible for selection. We selected a random sample of 500 patient records meeting the following inclusion criteria: 1) females 12 years and older, 2) had ever been prescribed hormonal contraception pills; and 3) ENDS documentation in the progress notes matching one the following keywords: “e-cig,” “ecig,” “e cig,” “vape,” “vaping,” “juul,” and “jool”, consistent with prior research (Young-Wolff et al., 2017). We searched for these terms in both structured and free-text fields. In the EHR system we examined, structured tobacco history was divided into two major components: “smoking status” and “smokeless tobacco use.” “Smoking status” can be expanded upon using a checklist with the options, “cigarette,” “pipe,” “cigar,” “e-cigarette,” and “vape”. Providers may also clarify tobacco use in a “comment” field. Neither the checklist nor the comment field are required fields.

Two reviewers independently reviewed all patient records from June-September 2020. They abstracted information from the structured tobacco history section, along with any mention of ENDS in the free text of the progress notes. The reviewers recorded ENDS documentation, contraception prescription information, and patients’ thrombotic risk factors into a REDCap (Research Electronic Data Capture) database. Reviewers captured free text from notes to categorize the nature of the ENDS documentation. Coding conflicts were resolved by consensus. This research was approved by the university’s Institutional Review Board.

## 3 Results

Of the 500 patient records, 28 were excluded because they did not have ENDS documentation. Of the remaining records, 245 (51.9%) patients reported ENDS use, which was documented either in the structured tobacco history or free text of progress notes. The remaining 227 (48.1%) patients were non-ENDS users whose record had an ENDS keyword match (e.g., patient information sheet advising the patient to refrain from using ENDS prior to surgery). The sample of ENDS users was predominately white, non-Hispanic, and about half were documented as never smokers in structured EHR fields (Table 1). Of the 245 ENDS users, 91 (37.1%) had no documentation of ENDS use in the structured tobacco history.

**Table 1:** Sample characteristics, by electronic nicotine delivery systems (ENDS) use status.

	ENDS user(N = 245)	Non-user(N = 227)	Overall(N = 472)
Age, in years			
12–17	8 (3.3%)	17 (7.5%)	25 (5.3%)
18–24	93 (38.0%)	64 (28.2%)	157 (33.3%)
25–34	96 (39.2%)	79 (34.8%)	175 (37.1%)
35 or older	47 (19.2%)	67 (29.5%)	114 (24.2%)
Race			
Asian	9 (3.7%)	3 (1.3%)	12 (2.5%)
Black or African American	20 (8.2%)	61 (26.9%)	81 (17.2%)
White	204 (83.3%)	146 (64.3%)	350 (74.2%)
Other	12 (4.9%)	14 (6.2%)	26 (5.5%)
Ethnicity			
Hispanic	18 (7.3%)	22 (9.7%)	40 (8.5%)
Not Hispanic	227 (92.7%)	202 (89.0%)	429 (90.9%)
Insurance status			
Medicare/Medicaid	68 (27.8%)	73 (32.2%)	141 (29.9%)
Private insurance	138 (56.3%)	131 (57.7%)	269 (57.0%)
Uninsured	29 (11.8%)	8 (3.5%)	37 (7.8%)
Other	10 (4.1%)	15 (6.6%)	25 (5.3%)
Smoking status			
Current smoker	92 (37.6%)	7 (3.1%)	99 (21.0%)
Former smoker	99 (40.4%)	38 (16.7%)	137 (29.0%)
Never smoker	53 (21.6%)	180 (79.3%)	233 (49.4%)
Never assessed	1 (0.4%)	2 (0.9%)	3 (0.6%)

Excluded 28 cases that did not have ENDS documentation; 1 patient record was missing age; 3 patient records were missing race and ethnicity.

Among the ENDS users, there were 82 contraception-related encounters with ENDS documentation. The nature of ENDS documentation in encounters related to contraception is summarized in Table 2, with 55 records (67.1%) only documenting that the patient was using ENDS. In 17 (20.7%) records, the provider counseled against ENDS use. Within this group, three OB-GYNs refused to prescribe CHC for patients using ENDS. Six notes (7.3%) included patient hand-outs forms advising to refrain from using e-cigarettes when using contraception. The remaining 4 notes (4.9%) did not fall into any of these categories. Of note, one of these records documented a pulmonary embolism in an emergency department patient who was prescribed CHC and used ENDS.

**Table 2:** Electronic nicotine delivery systems (ENDS) documentation in encounters related to contraception, by ENDS use status.

	Documentation for ENDS users (n = 82)^1^				Documentation for non-users (n = 43)^2^		
	ENDS use status assessed	Provider counseled against ENDS use	Contraception information form instructing patient to avoid ENDS	Other	ENDS use status assessed	Contraception information form instructing patient to avoid ENDS	Other
Records, n (%)	55 (67.1%)	17 (20.7%)	6 (7.3%)	4 (4.9%)	35 (81.4%)	3 (7.0%)	5 (11.6%)
Setting							
OB-GYN	11 (20.0%)	7 (41.2%)	2 (33.3%)	0	1 (2.9%)	2 (66.7%)	0
Pediatrics	17 (30.9%)	1 (5.9%)	1 (16.7%)	1 (25.0%)	33 (94.2%)	0	3 (60.0%)
Family Medicine	20 (36.4%)	4 (23.5%)	0	2 (50.0%)	1 (2.9%)	1 (33.3%)	2 (40.0%)
Internal Medicine	3 (5.5%)	1 (5.9%)	0	0	0	0	0
Other	4 (7.3%)	4 (23.5%)	3 (50.0%)	1 (25.0%)	0	0	0

1 Total number of encounters with ENDS documentation in ENDS users = 471; 82 encounters related to contraception with ENDS documentation (59 patients); 389 encounters unrelated to contraception with ENDS documentation (185 patients)

2 Total number of encounters with ENDS documentation in non-users = 317; 43 encounters related to contraception with ENDS documentation (41 patients); 274 encounters unrelated to contraception with ENDS documentation (186 patients).

Among the ENDS non-users, there were 43 contraception-related encounters with ENDS documentation. In 35 records (81.4%), the provider documented the patient does not use ENDS; 94.2% of these notes were from pediatricians. Three records contained the patient hand-outs described previously. Two records documented patients were considering switching from cigarettes to ENDS. Three records documented pediatric patients’ second-hand exposure to ENDS.

## 4 Discussion

ENDS use is under-documented in encounters related to contraception. Among 245 ENDS users, only 24% had documentation of ENDS use in encounters related to contraception prescriptions. These patterns are at least partially influenced by suboptimal ENDS documentation in the EHR. Several studies have revealed under-documentation of ENDS use in EHRs based on various types of encounters (LeLaurin et al., 2020, Winden et al., 2015, Young-Wolff et al., 2017). One study found there were exponential increases in ENDS documentation from 2006 to 2015, but in absolute terms, only a small portion of ENDS use was documented in the EHR (Young-Wolff et al., 2017). Because of structured fields in EHRs, cigarette smoking is documented more consistently than ENDS use. In fact, a Cochrane review found that when EHR modifications were made to prompt providers, documentation of cigarette smoking and smoking cessation counselling both increased (Boyle et al., 2014).

Although ENDS documentation is low in encounters related to contraception, one point that should be highlighted is that in this study, just under half of the records with ENDS documentation were from patients who have never used ENDS. This may reflect a trend towards increased screening among providers.

In the EHR system we examined, one feature that contributed to low rates of ENDS documentation is that the system did not require providers to indicate the “type” of tobacco product used when a patient is identified as a tobacco user. Providers also have an option to specify the type of tobacco in a “comment” field, but this is often left blank as well. Among 245 ENDS users, 91 (37%) had no mention of ENDS use in the structured tobacco history. Making the “types” field required would help clarify the ENDS-use status of many patients.

Another issue surrounding ENDS documentation was apparent confusion as to whether ENDS use should be documented under “smoking status” or “smokeless tobacco use”. While we found that most ENDS users were classified as current or former smokers (78.0%), there were also several ENDS users classified as never smokers (22.0%). Additionally, there were 41 ENDS users (16.7%) documented as users of smokeless tobacco. It is unclear whether the providers intended to record that these patients were using smokeless tobacco products such as chewing tobacco, or if they considered ENDS a type of smokeless tobacco product. Provider education on current trends in tobacco use and enhancements to EHR templates can help improve the accuracy of this documentation. Furthermore, providers need to be clear with patients when they are screening for tobacco use, as there seems to be confusion about the classification of ENDS among patients as well. In a focus group of pediatric providers, one provider emphasized the importance of using accessible language with patients, explaining “Unless you ask specifically— ‘Do you do vaping?’—they don’t answer” (LeLaurin et al., 2020). Another provider explained that some patients do not realize the liquid in ENDS cartridges contains nicotine (LeLaurin et al., 2020). This highlights the critical need for providers to ask clear and specific questions when screening for ENDS use.

Under-documentation of ENDS use in contraception-related encounters could be attributable to lack of clarity in the EHR. However, this under-documentation may also be related to providers’ perception of the risks of using ENDS on CHC. Among the 82 records with ENDS documentation in encounters related to contraception, providers only explicitly counseled against ENDS use in 17 records. This may reflect that many providers do not believe there is a significant risk associated with using ENDS on CHC. Future studies should explore providers’ perspectives on this matter. The emerging literature on the cardiovascular effects of ENDS is limited, as most studies have only shown acute elevations in biomarkers that suggest increased cardiovascular risks. For many patients, the benefits of contraception certainly outweigh potential risks of using ENDS while taking CHC. However, incorporating ENDS use into tobacco screening is a simple intervention on the part of the provider that can possibly help minimize any potential risks.

This exploratory study was conducted in a single academic healthcare system, limiting generalizability to other patients, healthcare providers, and EHR systems. Additionally, the inclusion of records was based on a keyword match of common ENDS terms consistent with prior research on ENDS documentation in EHRs (Young-Wolff et al., 2017). It is possible some documentation of ENDS use was missed by the algorithm. Additionally, this study included all records prior to July 2020 that met the described inclusion criteria. Providers’ documentation habits may have changed over the course of several years. This study did not examine how the documentation rates have changed over time. A larger scale EHR review should be conducted to make more generalizable conclusions.

## 5 Conclusion

To our knowledge, this is the first study that examines ENDS documentation in healthcare visits related to contraception. We found ENDS use is under-documented in contraception-related encounters, and the rate of counseling against ENDS use is even lower. Low documentation rates are likely related to suboptimal EHR prompts for tobacco screening, as well as a lag between current trends in ENDS use and provider documentation practices. These results illustrate the need to improve EHR prompts and increase provider awareness to standardize ENDS documentation. Further, this study highlights the need for providers to ask clear and specific questions when screening for tobacco use. Incorporating ENDS use into tobacco screening is a simple intervention with potential benefits.

## 6 Declarations

### Funding

This work was supported in part by the Florida Department of Health, grant number 9JK05. The funder had no role in study design, data collection and analysis, decision to publish, or preparation of the manuscript.

### 6.2 Ethics Approval

This study was approved by the University of Florida Institutional Review Board.

#### CRediT authorship contribution statement

**Joanna M. Theophilopoulos:** Conceptualization, Formal analysis, Investigation, Data curation, Writing – original draft. **Jennifer H. LeLaurin:** Formal analysis, Data curation, Writing – original draft. **Maribeth Williams:** Writing – review & editing. **Melissa Bright:** Writing – review & editing. **Lindsay A. Thompson:** Conceptualization, Writing – review & editing. **Ramzi G. Salloum:** Conceptualization, Data curation, Writing – original draft.

## Declaration of Competing Interest

The authors declare that they have no known competing financial interests or personal relationships that could have appeared to influence the work reported in this paper.
